# “I Feel Happy Again”: Methylphenidate Supports Health-Related Quality of Life in Survivors of Pediatric Brain Tumor

**DOI:** 10.3390/children9071058

**Published:** 2022-07-15

**Authors:** Sarah J. Verity, Lauren Bell, Jade Ryles, Rebecca M. Hill

**Affiliations:** 1 Newcastle Upon Tyne Hospitals NHS Foundation Trust, Royal Victoria Infirmary, Queen Victoria Road, Newcastle Upon Tyne NE1 4LP, UK; jade.ryles@nhs.net (J.R.); rebecca.hill@ncl.ac.uk (R.M.H.); 2 Northern Institute of Cancer Research, Newcastle University, Newcastle Upon Tyne NE1 4LP, UK; 3 Cumbria, Northumberland Tyne and Wear NHS Foundation Trust, Hopewood Park Hospital, Sunderland SR2 0NB, UK; lauren.bell2@cntw.nhs.uk; 4 Wolfson Childhood Cancer Research Centre, Newcastle University Centre for Cancer, Newcastle upon Tyne NE1 8QB, UK

**Keywords:** health-related quality of life, brain tumor, survivorship, cancer, children

## Abstract

Background: The deleterious impact upon the cognitive development of survivors of pediatric brain tumors (PBT) is well documented. Impairment in cognitive function is associated with reduced health-related quality of life (HRQoL), such that survivors of PBT report difficulties in multiple distinct domains and an overall reduced quality of life. Studies of the use of methylphenidate in survivors of PBT to alleviate impairment in cognitive functions have shown some success. The current study aimed to explore the impact upon HRQoL in survivors of PBT of a trial of psychostimulant medication. Method: Data were collected from 12 pediatric neuro-oncology patients aged 7–17 years receiving methylphenidate treatment. HRQoL was measured using the PEDS QL quality of life self-report measure and a semi-structured questionnaire-based interview. Results: Analyses of data demonstrates benefit to five domains associated with HRQoL: social, emotional, academic, physical, and cognition. Conclusion: Survivors of PBT reported favorable views as to the subjective benefit of methylphenidate on post-treatment impairment of HRQoL. This medication may offer the potential for restoration of a sense of ‘normality’ of function following cancer treatment in this clinical population.

## 1. Introduction

As survival rates increase in pediatric brain tumor, there is a growing awareness of the long-term neurocognitive and psychological correlates of survivorship [1,2]. Survivors of pediatric brain tumor (PBT) frequently show disrupted white matter development, reducing the rate at which individuals can process information from the world around them [3,4,5]. This slowed function affects all aspects of the quality of life of survivors. ‘Health-Related Quality of Life’ (HRQoL) refers to the combined experience of cognitive, emotional, physical, and social wellbeing related to health conditions [6]. HRQoL is reduced in survivors of PBT comparative to the healthy population and to survivors of other pediatric cancers [7,8]. Effective provision of survivorship support must include targeted interventions addressing the following factors associated with lowered HRQoL.

Approximately 50% of survivors of PBT show neurocognitive decline associated with white matter reduction [9]. Whilst deficit has been reported in multiple functions of cognition, the most significant impairment is shown in the processes involving cognitive processing skills such as attention, working memory, and processing speed [10]. Impairment in these domains is associated with lower academic attainment [11]. Survivors of pre-school brain tumors (<5 years at diagnosis) show a lower level of academic attainment compared to healthy controls and are substantially more likely to require special educational resources than peers with other types of cancer [12]. Academic performance is directly associated with HRQoL, with lowered academic attainment affecting outcomes such as subsequent employment, mental health, and life satisfaction [13].

In terms of social and emotional wellbeing, survivors of PBT experience fewer and less rewarding interpersonal relationships and report greater social isolation [14]. They are less likely to reach key interpersonal milestones such as independent living and long-term live-in relationships [15]. A cohort study of survivors of PBT found that 12% reported suicidal ideation, indicating a substantially increased risk compared to the overall population [16]. Increased social interaction is an identified protective factor in emotional wellbeing [17]; however, the difficulties in acquiring a stable friendship group associated with cognitive impairment produce a barrier to achieving this for survivors. Interventions that enhance social functionality in survivors of PBT are likely to increase HRQoL and long-term socio-emotional wellbeing in this group. The factors that support emotional wellbeing (peer relationships, social engagement, and academic success) are affected by the increased physical and cognitive fatigue commonly reported by survivors of PBT [18]. This ongoing hidden burden results in substantial cognitive and physical fatigue, such that survivors are less able to maintain peer relationships and to engage in out-of-school social activities.

### Intervention Targeting Neurocognitive Impairment

Methylphenidate offers a benefit in managing difficulties experienced in long-term survivorship following acquired brain injury, demonstrably reducing mental fatigue and social functioning difficulties [19,20,21,22]. Previous research in brain tumor cohorts identifies positive effects upon attention and processing speed following administration of methylphenidate in a mixed brain tumor/acute lymphoblastic leukaemia cohort [23,24,25]. A systematic review of the effect of methylphenidate on attention in the pediatric population shows benefit to selective and sustained attention [26]. Whilst the impact of methylphenidate on treatment-related neurocognitive impairment has been established, the indirect impact of neurocognitive intervention on HRQoL has not been investigated.

The goal of the current project was to assess the impact of methylphenidate specifically upon the HRQoL of a sample of patients with brain tumors. We hypothesized that participant ratings of HRQoL would increase following the use of methylphenidate.

## 2. Materials and Methods

### 2.1. Design

Data gathered during a recent clinical audit of current practice was used to consider the effect of methylphenidate on HRQoL. A mixed-method approach of data collection was employed in which results of a standardized psychometric patient-report questionnaire were explored by a semi-structured questionnaire and clinical interview.

### 2.2. Participants

Participants for the current study were recruited as part of a clinical audit from the wider sample of participants in our methylphenidate pilot study. Inclusion/exclusion criteria for the wider cohort screened for methylphenidate were as follows: Eligible participants were aged between 5.0 and 15.5 years at recruitment; had a general ability index ≥ 50; had a brain tumor/previous brain tumor; had completed initial treatment at least 12 months prior to baseline assessment for this study; and had either hydrocephalus at diagnosis or had received cranial radiotherapy. Eligible patients showed tumor/treatment-related cognitive impairment as measured by the processing speed index of the Wechsler Intelligence Scale for Children (version V). Exclusion criteria included all medical and psychological contraindications to methylphenidate hydrochloride in children.

**Figure 1:**
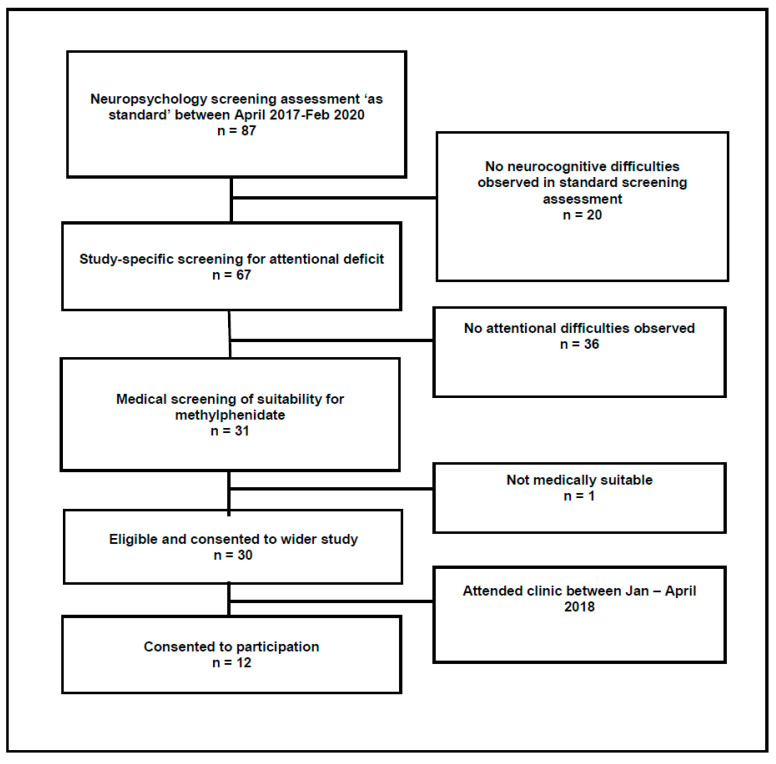
Flowchart of screening for eligibility.

A convenience sample of patients who attended the clinic between January and April 2018 were recruited to talk in more detail about their HRQoL (see Figure 1). One hundred percent of patients attending the clinic during the recruiting timeframe consented to share information about their experience of methylphenidate (*n* = 12). All patients had received methylphenidate for >4 weeks duration, the majority of whom had received methylphenidate for 6 months. Demographic characteristics of the participants are detailed in Table 1. Participants from the HRQoL sample did not differ from the wider methylphenidate clinic sample in mean age, presence/absence of hydrocephalus, level of general intellectual ability, or level of processing speed deficit.

### 2.3. Materials

HRQoL measurements were taken using two methods: the PEDS QL and the EMTQ.

#### 2.3.1. Pediatric Quality of Life Inventory (PedsQL™)

The PedsQL™ [27] is a multidimensional measure designed to assess the HRQOL of children. The questionnaire comprises 23 items measuring the physical, emotional, social, and life-function aspects of HRQOL. Items are scored from 0 (“Never a problem”) to 4 (“It is almost always a problem”), thus a higher score reflects a lower quality of life. Research demonstrates feasibly, reliability, and validity as pediatric population health outcomes; the Cronbach’s alpha is 0.89 for the child and 0.92 for the parent report. The PEDS_QL data were gathered prior to and following the use of methylphenidate (6 weeks ± 2 weeks).

#### 2.3.2. Experience of Methylphenidate Treatment Questionnaire (EMTQ)

The EMTQ is a custom-built questionnaire developed to assess the HRQoL of participants (Appendix A). Items for the EMTQ were identified by conducting an audit of the clinical notes of pediatric neuro-oncology patients using methylphenidate. Themes that were raised by patients or parents during clinical appointments over the six-month period prior to questionnaire development were considered for inclusion by a clinical psychologist and a nurse specialist working in pediatric neuro-oncology. Face validity of items was assessed through patient participant involvement, including review by two patient–parent dyads. Adjustments to the questionnaire to promote accessibility were made following the PPI review. Twelve questions were finalized to assess the impact of methylphenidate on aspects of HRQoL identified by our patient group: social life, perceived independence, mood, confidence, school life, self-esteem, interpersonal relationships, and fatigue levels. Face validity and patient acceptability were assessed.

### 2.4. Procedure

Immediate-release methylphenidate was prescribed on a starting dose of 2.5 mg twice daily for children 15–20 kg; 5 mg twice daily for those 21–30 kg, and 10 mg twice daily for those above 30 kg as per the British National Formulary (Child) [28]. Mean starting dose of methylphenidate stated as mg per kg was 0.19 (range (0.11–0.32). Optimal dose was identified by the increase in the child’s attentional scores towards their estimated premorbid ability whilst minimizing side effects. Mean optimal dose of methylphenidate was 0.34 mg/kg per twice-daily dose (range 0.2–0.67). Clinical assessment of height, weight, heart rate, and blood pressure was conducted following NICE guidelines [29].

All participants were provided with HRQoL questionnaires during a routine clinical appointment. Participants filled in the PEDS-QL and EMTQ questionnaire independently or received help from a clinical psychologist or parent if required. Participants were asked to expand upon their responses to the EMTQ by clinical interview within standard follow-up appointments.

### 2.5. Ethical Considerations

Ethical approval was obtained for this audit by the Newcastle University Ethics Committee. The audit was registered with Newcastle Upon Tyne Hospitals NHS Foundation Trust (Reg. No. 9049). Parents of participants provided verbal consent for data to be collected. Assent was sought from participants.

### 2.6. Data Analysis

Paired sample *t*-tests were performed upon mean scores at baseline and follow-up for child report data. For all statistical tests, SPSS 24 computerized statistical package was used [30].

Qualitative data were analyzed using thematic analysis, an analytic method that explores patterns and themes in qualitative data [31]. The participant’s responses were initially coded and then organized into potential sub-themes and overarching themes. These were next reviewed by an independent co-rater. From this, lower-order sub-themes were analyzed to provide a five-theme solution as the best fit for the data. Physical, emotional, social, academic, and neuropsychological impacts of methylphenidate were identified. Lower-order sub-themes, relating to each of the five higher-order themes were categorized. Data saturation was achieved as no additional themes were identified within the data [32]. Once themes had been established, a member check was completed with two participants.

## 3. Results

### 3.1. Quantitative Data

**Figure 2:**
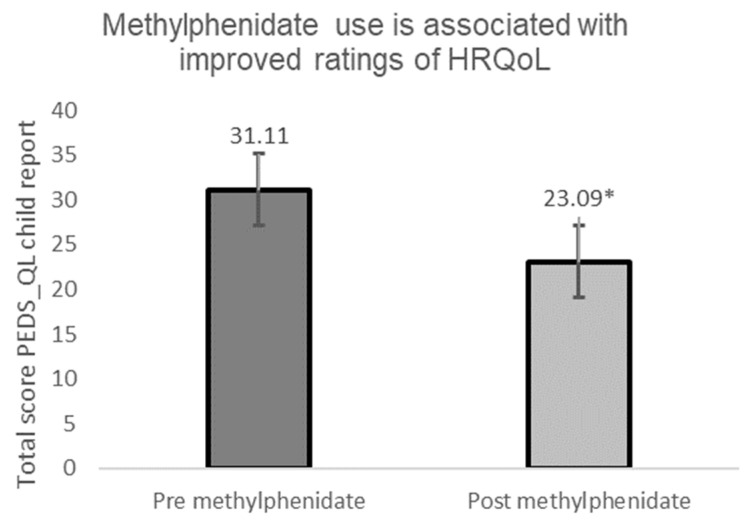
Pre versus post use of methylphenidate measurement of HRQoL. Lower HRQoL scores show fewer and/or less significant problems. * Significant at *p* ≤ 0.05.

Child report ratings of quality of life (QoL) as measured by the Pediatric Quality of Life Inventory showed a positive benefit of methylphenidate on QoL at 12 months: t (21) = 3.63, *p* = 0.02, d = 0.54. Results of the overall increase in reported function are shown in Figure 2.

Analyses of the separate components of quality of life measured by the PEDS_QL found a statistically significant increase in physical function, emotional function, and social function. The results of these are shown in Table 2. Interestingly, a statistically significant change was not found in reports of school (academic) function.

Consistent with the corresponding semi-structured questionnaire responses, a significant difference was reported between the mean HRQoL scores following intervention in the following areas: Physical function (t (17) = 2.98, *p* ≤ 0.01); emotional function (t (17) = 3.12, *p* ≤ 0.01); and social function (t (18) = 2.19, *p* = 0.04). No significant difference was found in perceived school (academic) function as measured by the PEDS-QL.

### 3.2. Qualitative Data

**Figure 3:**
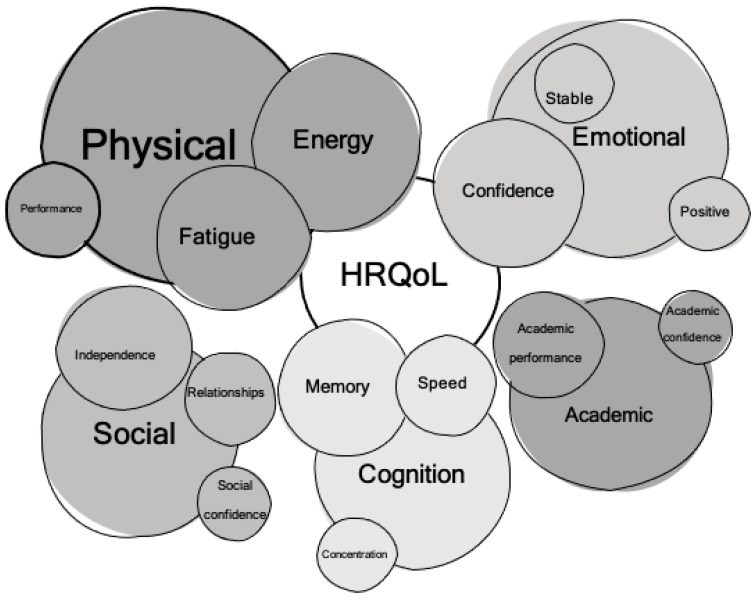
Thematic map of identified themes related to health-related quality of life (HRQoL) in pediatric neuro-oncology patients. Larger circles denote a greater level of endorsement of benefit in that domain, smaller areas denote a relatively lesser level of endorsement.

Coding of the data suggested five overarching themes related to methylphenidate use: Physical, social, academic, neuropsychological/cognitive, and emotional effects. Lower order themes were organized within these five overarching themes. Themes are represented as Figure 3.

### 3.3. Physical Impact

Most participants (11/12 participants) referred to the physical impact of tumor and treatment on their quality of life, reporting positive changes to physical wellbeing on methylphenidate treatment. Key themes addressed by participants pertaining to physical changes following methylphenidate treatment were: Fatigue prior to methylphenidate; energy levels following methylphenidate; and physical performance. One of the most frequently reported difficulties faced prior to the prescription of methylphenidate was fatigue: “*I got tired very easily and sometimes found it difficult to concentrate and keep up in lessons at school*”—Participant 1. Following the treatment of methylphenidate, the most common report (9/12 participants) was an improvement in energy levels: “*I used to come home from work or school and be so tired I had to go to bed. Now, I can stay awake the whole day.*”—Participant 4.

### 3.4. Emotional Impact

Participants (10/12 participants) reported at least one emotional impact resulting from taking methylphenidate. Three sub-themes were identified from the data: ‘positive emotions’, ‘emotional stability’, and ‘self-confidence’. Three-quarters of the participants referred to an increase in positive emotions following the prescription of methylphenidate: “*I feel better and happy, less sad and poorly.*”—Participant 2. Many participants (7/12 participants) reported a benefit to their self-esteem of self-confidence of methylphenidate: “*[I feel] more confident in myself*”—Participant 11. Half of the participants referred to feeling less reactive to negative emotions and less affected by their environment. These instances were categorized as ‘emotional stability’: “*(I am) less sensitive- I don’t cry as easily now*”—Participant 7.

### 3.5. Social Impact

The social/interpersonal impact of methylphenidate on participants was reported by 8/12 participants. This theme was highlighted solely by participants over the age of 10 years. Categories identified were labeled ‘independence’, ‘relationships’, and ‘social confidence’. Following the prescription of methylphenidate 7/12 participants reported they felt able to be more independent in at least one aspect of their life: “*I don’t need to ask for help as much*”—Participant 8. Half of the participants referred to an improvement in their social relationships: “*My relationships are much better with my friends, family and school teachers*”—Participant 9. A third of the participants felt that their social confidence had improved on the use of methylphenidate: “*I am more confident when speaking with unknown people, friends and adults*”—Participant 4.

### 3.6. Academic Impact

Perceived changes to academic functioning were reported by 9/12 participants. Codes related to this theme were divided into two categories: ‘academic performance’ and ‘academic confidence’. Over half of the participants referenced an improvement in their academic performance: “*[My] grades went up to a much better standard*”—Participant 11. A third of the participants reported an improvement in academic confidence after beginning methylphenidate treatment: “*I feel far more confident providing an input in class*”—Participant 6.

### 3.7. Cognition Impact

Participants reported changes in function following methylphenidate treatment that relate to cognitive function. These were labeled ‘concentration’, ‘processing speed’, and ‘memory’. Nine participants reported an improvement in at least one aspect of concentration: “*I pay more attention to lessons and for the whole hour… I used to be easily distracted”*—Participant 4. Following treatment by methylphenidate, 6/12 participants referred to a perceived positive impact upon memory: “*I started (to) remember stuff from lessons earlier in the week*”—Participant 12.

## 4. Discussion

Survivors of PBT commonly experience significant adverse effects upon HRQoL. The current study aimed to explore the subjective experiences of survivors of PBT in response to methylphenidate treatment of cognitive late effects of tumor and treatment. Five overarching themes were identified from the data, highlighting the impact of methylphenidate on the physical, emotional, social, academic, and neuropsychological wellbeing of pediatric patients. Eleven out of twelve participants reported perceived positive changes to their HRQoL of methylphenidate.

### 4.1. Reduction of Fatigue Has the Greatest Impact on HRQoL in Survivors

Fatigue is the most commonly reported cancer-related symptom, with survivors of PBT identifying this to be the most distressing long-term effect of a brain tumor [33,34]. Survivors are found to be significantly more affected by fatigue than the general population, irrespective of the length of time since treatment. An association with HRQoL has been established, in which fatigue was found to be strongly associated with mood, socialization, cognitive function, and academic performance. Via these factors, fatigue was found to be the strongest predictor of poor HRQoL in survivors of PBT. Near consensus amongst participants (11/12) on the positive impact of methylphenidate upon physical sensations of fatigue was reported. Participants reported the alleviation of fatigue as effecting positive change on their sense of wellbeing, but also indirectly on other aspects of their lives. The reduction in fatigue at the end of the school day allowed participants to return to, or to take up, after-school activities that they had been previously obliged to miss due to exhaustion. In turn, engagement in out-of-school activities promoted social interaction with peer groups, leading to a stronger sense of community membership and social acceptance.

Survivors of pediatric cancer are less likely to engage in social activities and have fewer friendships than age-matched peers. Whilst satisfying interpersonal relationships correlate highly with high HRQoL in childhood cancer survivors, it appears that fatigue may be a contributory factor in preventing survivors of PBT from engagement in normal levels of social activity. The current study found that, following the prescription of methylphenidate, patients reported higher engagement with social activities, but also perceived an improvement in social relationships. The direct and indirect effects upon HRQoL of fatigue reduction suggest that the assessment and management of post-cancer fatigue should be a key target for clinicians in pediatric oncology services.

### 4.2. Reduction in Processing Speed Deficit via Methylphenidate Has a Positive Impact on Social Wellbeing

In addition to the positive benefit of social interaction on the alleviation of fatigue, the current study identified a relationship between speed of processing on perceived social ability. Participants’ reports of increased social activity were provided in the context of ‘not feeling too tired to go’ to after-school activities/friendship group outings, and also feeling ‘able to keep up’. Participants frequently alluded to feeling able to join in more to conversations and to respond quicker in social interactions (in the classroom, playground, and family environments). This finding is in line with research conducted on pediatric patients of traumatic brain injury (TBI) [34,35]. This group experience many difficulties shared with the PBT population, in which participants are reported to talk slower, take long pauses during conversation, and show difficulties in comprehending the responses of their social partner [35]. These difficulties lead to substantial social difficulties and reduced interpersonal efficacy. It is possible that the known positive impact of methylphenidate on processing speed effects positive changes in patients’ social competence via the increased ability to process information ‘in real time’. The findings of the current study suggest that whilst many aspects of PBT impairment are permanent, it may be possible to effect improvement in interpersonal function via improved speed of processing.

Unsurprisingly, the positive change to fatigue increased opportunities for social engagement, and support for interpersonal interaction of methylphenidate correlated with improved emotional wellbeing. The majority of participants reported feeling ‘happier’ since taking methylphenidate, perceived ‘happiness’ being a defining attribute of HRQoL. The increase in positive emotions may be due to the increase in social functioning. It is possible that the documented effect of methylphenidate on processing speed has a wider impact on social functionality, and this in turn leads to an improvement in overall HRQoL.

### 4.3. Methylphenidate Impacts Positively on Perceptions of Managing Schoolwork

Survivors of PBT experience substantial academic difficulties, requiring high levels of support in class and differentiated curricula and attaining reduced grades compared to peers and siblings. Slowed acquisition of academic gains in this group is attributable to the reduced ability to acquire new information at a comparable rate to age-matched peers. Participants of the current study reported perceived benefits to their academic functioning. It is likely that the impact of methylphenidate on processing speed is also involved in perceived academic gains. The current sample showed participants’ perception of increased processing speed (reported as ‘thinking quicker’, ‘being able to respond faster’, and ‘keeping up’). A study of the relationship between processing speed, fatigue, and academic attainment following methylphenidate treatment would be of interest.

The current study suggests that the experience of chronic high-level exhaustion may be a contributory factor to academic difficulties. An association between increased drowsiness with poorer academic performance in children has been established, as has an association between speed of processing and academic attainment. Participants reported a struggle to stay awake during lessons, describing chronic and unrelenting moderate-level fatigue over a period of months to years prior to treatment with methylphenidate. Mediational analysis of the impact of this treatment in a larger sample would allow exploration of the relationship between processing speed and fatigue with respect to rates of academic attainment. Furthermore, the relationship between fatigue, social interaction, and emotional wellbeing could bear further investigation, potentially allowing for a more targeted intervention of social difficulties subsequent to cancer treatment.

### 4.4. Strengths and Limitations of the Current Study

This study is the first to explore the subjective experience of methylphenidate in survivors of PBT. Whilst quality of life measures are successfully included in a number of children’s cancer trials, these gather quantitative data via self and parent report questionnaire measures such as the PEDS QL. Such questionnaires limit participants to reporting on only those aspects of HRQoL included in the questionnaire and may neglect other experiences. The current study was patient-led, with questionnaire items collated from the clinical feedback of families and patients, providing a high level of ecological validity, external validity, and reliability of this measure.

The current study provides very positive initial data on improvement in HRQoL in the PBT population following treatment with methylphenidate; however, we acknowledge methodological limitations. Given the proven benefit to survivors of PBT of methylphenidate, our center felt obliged to offer the treatment to all eligible children, thus leaving no control sample. Whilst demonstrable gains in HRQoL over the period of methylphenidate use are evident from the child’s baseline scores, the sudden reduction in fatigue and improvement in positive feelings of wellbeing may be attributable to an unknown factor. Further studies using a randomized control to assess HRQoL would greatly enhance existing evidence. Whilst our convenience sample provided sufficient pilot data to support an application for funding a larger study, the results of this small sample are indicative rather than generalizable. These limitations will be addressed in our larger multi-site UK trial: COGINT, opening in July 2022.

## 5. Conclusions

This study is the first to describe the impact of methylphenidate on HRQoL in survivors of PBT. Two summary points are provided. First, methylphenidate offers patient-perceived benefits to HRQoL in five key areas: physical wellbeing, emotional wellbeing, social function, academic ability, and cognitive function. Second, the impact of fatigue upon HRQoL emerged as a key variable affecting social, emotional, and academic domains. The direct and indirect results of fatigue upon improved HRQoL suggest that fatigue management should be a priority for pediatric neuro-oncologists in long-term follow-up. Methylphenidate treatment of survivors of PBT has the potential to benefit HRQoL in this group, providing survivors with a sense of regained ‘normality’ following cancer treatment.

## Figures and Tables

**Table 1:** Demographic and clinical characteristics of the sample.

	*n*	%	
**Sex**			
Female	4	33.3	
Male	8	66.7	
**Ethnicity**			
White British	12	100	
**Diagnosis**			
Medulloblastoma	2	16.7	
Ependymoma	3	25	
Low-grade glioma	5	41.7	
Atypical teratoid/rhabdoid tumor	1	8.3	
Pineoblastoma	1	8.3	
Total	12	100	
**Tumor location**			
Infratentorial tumor	7	58.3	
Supratentorial tumor	5	41.7	
**CSF status**			
No hydrocephalus	3	25	
Hydrocephalus present	9	75	
**Radiotherapy**			
Cranial radiotherapy	8	66.7	
No radiotherapy	4	33.3	
**Age at diagnosis (years)**	**Mean**	**SE**	**Range**
Mean	7.08	1.38	1–16
**Years since diagnosis (years)**	**Mean**	**SE**	**Range**
Mean	6.2	0.99	1–12
**Age commencing MPH (years)**	**Mean**	**SE**	**Range**
Mean	13.3	0.95	7–17
**IQ**	**Mean**	**SE**	**Range**
Verbal IQ (WISC IV VCI)	94	3.4	73–114
Perceptual IQ (WISC IV PRI)	96	2.5	88–104
Working memory (WISC IV WMI)	95	2.8	82–104
Processing speed (WISC IV PSI)	76.5	2.7	70–91

Notes: CSF—cerebrospinal fluid, IQ—intellectual quotient, WISC IV—Wechsler Intelligence Scale for Children version IV, SE—standard error.

**Table 2:** Pre- to post-intervention scores of HRQoL function.

PEDS-QL Domain	Mean	SD	t (df)	Sig.	95% CI of the Difference	
					Lower	Upper
Physical function	2.61	3.71	2.98 (17)	0.01 *	0.77	4.46
Emotional function	2.5	3.4	3.12 (17)	0.01 *	0.81	4.2
Social function	2.21	4.4	2.19 (18)	0.04 *	0.09	4.3
School function	1.11	3.59	1.34 (18)	0.2	−0.62	2.84

* Significance at *p* =< 0.05. Notes: SD = standard deviation; CI = confidence interval.

## Data Availability

The data presented in this study are available on request from the corresponding author in redacted form. The data are not publicly available to preserve anonymity of participants.

## Appendix A

### Experience of Methylphenidate Treatment Questionnaire

Thank you for taking the time to answer the following questions. Please aim to expand on your answers in order to provide as much information as possible. You are welcome to discuss this with your parents, and for your parents to write down the answers for you, as long as it is your own words.

We will then discuss with you your answers in your next clinic appointment. We might ask you to give us a bit more information about some of your answers, if you can.

1. What were the biggest difficulties in your life before taking methylphenidate?
2. How did you know when the methylphenidate started working (if it did)?
3. What were the first things you noticed?
4. How did you notice these things?
5. Has methylphenidate had an impact on your social life, and if so how?
6. Has methylphenidate had an impact on your independence, and if so how?
7. Has methylphenidate had an impact on your mood, and if so, how?
8. Has methylphenidate affected your confidence, and if so how?
9. Has methylphenidate impacted upon your school life, and if so how?
10. Has methylphenidate changed anything about how you feel about yourself, and if so how?
11. Has methylphenidate had an impact on your relationships, and if so how?
12. Has methylphenidate affected your fatigue levels, and if so how?

## References

[1] Stavinoha P.L., Askins M.A., Powell S.K., Smiley N.P., Robert R.S. (2018). Neurocognitive and Psychosocial Outcomes in Pediatric Brain Tumor Survivors. Bioengineering.

[2] Oyefiade A., Paltin I., De Luca C.R., Hardy K.K., Grosshans D.R., Chintagumpala M., Mabbott D.J., Kahalley L.S. (2021). Special Series: Neurocognitive Outcomes In Survivors Of Pediatric Cancer Review Articles Cognitive Risk in Survivors of Pediatric Brain Tumors. https://www.ncbi.nlm.nih.gov/pmc/articles/PMC8260914/.

[3] Oyefiade A., Beera K., Moxon-Emre I., Skocic J., Bartels U., Laughlin S., Ramaswamy V., Mabbott D. (2020). QOL-09. whole-brain white matter network connectivity is disrupted by pediatric brain tumor treatment. Neuro Oncol..

[4] Wier R., Aleksonis H.A., Pearson M.M., Cannistraci C.J., Anderson A.W., Kuttesch J.F., Compas B.E., Hoskinson K.R. (2019). Fronto-limbic white matter microstructure, behavior, and emotion regulation in survivors of pediatric brain tumor. J. Neuro Oncol..

[5] Peterson R.K., Tabori U., Bouffet E., Laughlin S., Liu F., Scantlebury N., Mabbott D. (2019). Predictors of neuropsychological late effects and white matter correlates in children treated for a brain tumor without radiation therapy. Pediatr. Blood Cancer.

[6] Karimi M., Brazier J. (2016). Health, Health-Related Quality of Life, and Quality of Life: What is the Difference?. Pharmacoeconomics.

[7] Michel G., Brinkman T.M., Wakefield C.E., Grootenhuis M. (2020). Psychological Outcomes, Health-Related Quality of Life, and Neurocognitive Functioning in Survivors of Childhood Cancer and Their Parents. Pediatr. Clin. N. Am..

[8] Aukema E.J., Netteke A.Y., Meeteren S.-V., Last B.F., Maurice-Stam H., Grootenhuis M.A. (2013). Childhood Brain Tumor Survivors at Risk for Impaired Health-Related Quality of Life. www.jpho-online.com.

[9] Askins M.A., Moore B.D. (2008). Preventing neurocognitive late effects in childhood cancer survivors. J. Child Neurol..

[10] Palmer S.L., Armstrong C., Onar-Thomas A., Wu S., Wallace D., Bonner M.J., Schreiber J., Swain M., Chapieski L., Mabbott D. (2013). Processing speed, attention, and working memory after treatment for medulloblastoma: An international, prospective, and longitudinal study. J. Clin. Oncol..

[11] Schreiber J.E., Gurney J.G., Palmer S.L., Bass J.K., Wang M., Chen S., Zhang H., Swain M., Chapieski M.L., Bonner M.J. (2014). Examination of risk factors for intellectual and academic outcomes following treatment for pediatric medulloblastoma. Neuro Oncol..

[12] Stavinoha P.L., Trinh-Wong T., Rodriguez L.N., Stewart C.M., Frost K. (2021). Educational Pain Points for Pediatric Brain Tumor Survivors: Review of Risks and Remedies. Children.

[13] Remes T.M., Hovén E., Ritari N., Pohjasniemi H., Puosi R., Arikoski P.M., Arola M.O., Lähteenmäki P.M., Lönnqvist T.R.I., Ojaniemi M.K. (2021). Neurocognitive impairment, employment, and social status in radiotherapy-treated adult survivors of childhood brain tumors. Neuro Oncol. Pract..

[14] Vannatta K., Gartstein M.A., Short A., Noll R.B. (1998). A controlled study of peer relationships of children surviving brain tumors: Teacher, peer, and self ratings. J. Pediatr. Psychol..

[15] Kunin-Batson A., Kadan-Lottick N., Zhu L., Cox C., Bordes-Edgar V., Srivastava D.K., Zeltzer L., Robison L.L., Krull K.R. (2011). Predictors of independent living status in adult survivors of childhood cancer: A report from the Childhood Cancer Survivor Study. Pediatr. Blood Cancer.

[16] Brinkman T.M., Liptak C.C., Delaney B.L., Chordas C.A., Muriel A.C., Manley P.E. (2013). Suicide ideation in pediatric and adult survivors of childhood brain tumors. J. Neuro Oncol..

[17] Zeltzer L.K., Lu Q., Leisenring W., Tsao J.C.I., Recklitis C., Armstrong G., Mertens A.C., Robison L.L., Ness K.K. (2008). Psychosocial outcomes and health-related quality of life in adult childhood cancer survivors: A report from the Childhood Cancer Survivor Study. Cancer Epidemiol. Biomark. Prev..

[18] Irestorm E., Ora I., Linge H., Olsson I.T. (2021). Cognitive Fatigue and Processing Speed in Children Treated for Brain Tumours.

[19] Huang C.-H., Huang C.-C., Sun C.-K., Lin G.-H., Hou W.-H. (2016). Methylphenidate on Cognitive Improvement in Patients with Traumatic Brain Injury: A Meta-Analysis. Curr. Neuropharmacol..

[20] Manktelow A.E., Menon D.K., Sahakian B.J., Stamatakis E.A. (2017). Working memory after traumatic brain injury: The neural basis of improved performance with methylphenidate. Front. Behav. Neurosci..

[21] McDonald B., Flashman L., Arciniegas D., Ferguson R., Harezlak J., Xing L., Sphrehn G., Maerlender A., Saykin A., McAllister T. (2016). Cognitive therapy with and without methylphenidate after traumatic brain injury (TBI): Which is better?. Brain Inj..

[22] Johansson B., Wentzel A.-P., Andréll P., Mannheimer C., Rönnbäck L. (2015). Methylphenidate reduces mental fatigue and improves processing speed in persons suffered a traumatic brain injury. Brain Inj..

[23] Conklin H.M., Khan R.B., Reddick W.E., Helton S., Brown R., Howard S.C., Bonner M., Christensen R., Wu S., Xiong X. (2007). Acute neurocognitive response to methylphenidate among survivors of childhood cancer: A randomized, double-blind, cross-over trial. J. Pediatr. Psychol..

[24] Conklin H.M., Helton S., Ashford J., Mulhern R.K., Reddick W.E., Brown R., Bonner M., Jasper B.W., Wu S., Xiong X. (2010). Predicting methylphenidate response in long-term survivors of childhood cancer: A randomized, double-blind, placebo-controlled, crossover trial. J. Pediatr. Psychol..

[25] Conklin H.M., Reddick W.E., Ashford J., Ogg S., Howard S.C., Brown E.B.M., Bonner M., Christensen R., Wu S., Xiong X. (2010). Long-term efficacy of methylphenidate in enhancing attention regulation, social skills, and academic abilities of childhood cancer survivors. J. Clin. Oncol..

[26] Hagan A.J., Verity S.J. (2022). Translating methylphenidate’s efficacy on selective and sustained attentional deficits to those reported in childhood cancer survivors: A qualitative review. Appl. Neuropsychol. Child.

[27] Varni J.W., Burwinkle T.M., Seid M., Skarr D. (2003). The PedsQL^TM^* 4.0 as a Pediatric Population Health Measure: Feasibility, Reliability, and Validity. Ambul. Pediatr..

[28] Joint Formulary Committee (2022). British National Formulary for Children.

[29] (2018). Attention Deficit Hyperactivity Disorder: Diagnosis and Management NICE Guideline. www.nice.org.uk/guidance/ng87.

[30] (2016). IBM SPSS Statistics for Windows.

[31] Braun V., Clarkee V. (2006). Using thematic analysis in psychology. Qual. Res. Psychol..

[32] Saunders B., Sim J., Kingstone T., Baker S., Waterfield J., Bartlam B., Burroughs H., Jinks C. (2018). Saturation in qualitative research: Exploring its conceptualization and operationalization. Qual. Quant..

[33] Wood J., Verity S.J. (2021). Literature Review Exploring Evidence of Fatigue in Survivors of Pediatric Brain Tumors A Systematic Review. Cancer Care Res. Online.

[34] Shultz E.L., Hoskinson K.R., Keim M.C., Dennis M., Taylor H.G., Bigler E.D., Rubin K.H., Vannatta K., Gerhardt C.A., Stancin T. (2016). Adaptive functioning following pediatric traumatic brain injury: Relationship to executive function and processing speed. Neuropsychology.

[35] Struchen M.A., Sherer M., Sander A. (2014). Social communication interventions. Handbook on the Neuropsychology of Traumatic Brain Injury.

